# Shared governance in the plant holobiont and implications for one health

**DOI:** 10.1093/femsec/fiae004

**Published:** 2024-02-13

**Authors:** Gabriele Berg, Cristina Dorador, Dilfuza Egamberdieva, Joel E Kostka, Choong-Min Ryu, Birgit Wassermann

**Affiliations:** Institute of Environmental Biotechnology, Graz University of Technology, Petersgasse 12/I, 8010 Graz, Austria; Leibniz Institute for Agricultural Engineering and Bioeconomy (ATB), Max-Eyth-Allee 100, 14469 Potsdam, Germany; Institute for Biochemistry and Biology, University of Potsdam, Karl-Liebknecht-Str. 24-25, 14476 Potsdam, Germany; Department of Biotechnology, Universidad de Antofagasta & Centre for Biotechnology and Bioengineering (CeBiB), Angamos 601, Antofagasta, Chile; Institute of Fundamental and Applied Research, National Research University, TIIAME, Kari Niyazi street 39, Tashkent 100000, Uzbekistan; Medical School, Central Asian University, Milliy bog street 264, Tashkent 111221, Uzbekistan; Schools of Biological Sciences and Earth & Atmospheric Sciences, Center for Microbial Dynamics and Infection, Georgia Institute of Technology, 310 Ferst Drive, Atlanta, GA 30332, United States; Biosystems and Bioengineering, University of Science and Technology KRIBB School, 125 Gwahangro, Yuseong, Daejeon 34141, South Korea; Molecular Phytobacteriology Laboratory, Infectious Disease Research Center, KRIBB, 125 Gwahangro, Yuseong, Daejeon 34141, South Korea; Institute of Environmental Biotechnology, Graz University of Technology, Petersgasse 12/I, 8010 Graz, Austria

**Keywords:** holobiont, microbiome, one health, plant microbiome, symbiosis

## Abstract

The holobiont Holobiont theory is more than 80 years old, while the importance of microbial communities for plant holobionts was already identified by Lorenz Hiltner more than a century ago. Both concepts are strongly supported by results from the new field of microbiome research. Here, we present ecological and genetic features of the plant holobiont that underpin principles of a shared governance between hosts and microbes and summarize the relevance of plant holobionts in the context of global change. Moreover, we uncover knowledge gaps that arise when integrating plant holobionts in the broader perspective of the holobiome as well as *one* and planetary health concepts. Action is needed to consider interacting holobionts at the holobiome scale, for prediction and control of microbiome function to improve human and environmental health outcomes.

## Teaser

Plant holobiont research catalyzes new approaches for human and environmental health on a rapidly changing world.

## Introduction

In 1943, the holobiont theory was already empirically formulated by the German theoretical biologist Adolf Meyer-Abich. In 1991, the famous symbiosis researcher Lynn Margulis defined the holobiont again, and obviously independently of this first description, with the holobiont representing an association of partners (bionts) throughout a significant portion of the life history (Margulis 1991). After the merging of holobiont theory into the hologenome theory of evolution by Zilber-Rosenberg and Rosenberg (2008), the term has been used more widely and successively applied to different organisms, including plants (Vandenkoornhuyse et al. 2015). In the last two decades, microbiome research confirmed not only the holobiont theory, it substantially contributed to better insights into host–microbiota relationships and metabolite interplay (Gilbert et al. 2016, Cordovez et al. 2019, Berg et al. 2020). The microbiota, which can form diverse microbiomes in one host, consist of billions of microbial cells from all domains of life (Bacteria, Archaea, and Eukaryotes: Fungi, and Protists) (Berg et al. 2020). Each plant constitutes an individual holobiont comprised of multiple microbiomes that establish in a plant's above- and below-ground tissues. The different physio-chemical conditions in those tissues shape the microbiota and form the phyllosphere and rhizosphere (Philippot et al. 2013, Cordovez et al. 2019). More than a century ago, Lorenz Hiltner discovered the importance of rhizosphere-associated microorganisms for plant growth and health and described all principles of the holobiont concept for plants, however, without mentioning the term (Hartmann et al. 2008). The rhizosphere describes the soil—plant interface and is crucial for plant microbiome assembly and functioning of the plant holobiont (Berg and Smalla 2009). The rhizosphere serves as a nexus of communication and metabolic exchange between the plant and the surrounding soil environment. Here, we highlight how evolution of the plant holobiont have resulted in a shared governance of host and microbiota. Moreover, we address conclusions and knowledge gaps that arise from the broader perspective of one and planetary health.

## Evolution and ecology of plant holobionts

Plant evolution is driven in a large part by ancient microbial friends and recent foes (Delaux and Schornack 2021). The theory that specific arbuscular mycorrhizal fungi symbionts were drivers of plant terrestrialization in early Palaeozoic land ecosystems, 500–450 million years ago, is well-established but less is known about plant—bacteria interactions during evolution (Brundrett 2002). Nonvascular plants such as liverworts, hornworts, and mosses (bryophytes), the sister lineage to vascular plants, belonged to the first land colonizers (McDaniel 2021). Recently it was discovered that they harbour a more diverse but less specific microbiome compared to vascular plants (Wicaksono et al. 2021). While vascular plants recruit environmental bacteria through root exudates resulting in plant genotype-specific colonization, bryophytes lack functional roots and maintain their bacterial communities throughout their lifecycle. Mosses are known for close interaction with mycorrhizal fungi, yet their interaction with prokaryotic microorganisms seems to be highly important as well, as shown for *Funaria* (Hornschuh et al. 2006), *Sphagnum* (Bragina et al. 2012, 2014, Kostka et al. 2016), and *Riccia* (Wicaksono et al. 2023c).

The ecology of the plant holobiont is an important subject which is still not well-studied. Vertical transmission of the plant microbiota was only recently reported, for mosses via sporophytes (Bragina et al. 2012) and for spermatophytes via seeds (Adam et al. 2018, Johnston-Monje et al. 2016). However, both vertical and horizontal transmission ensure survival of offspring. Particularly the microbiome of seeds has gained scientific attention over the past years due to its benefits for the host plant (Berg and Raaijmakers 2018). A recent large-scale meta-study on the seed microbiomes of 50 plant species revealed a diverse and flexible seed microbiome including several high abundant core microbiota (Simonin et al. 2022). In general, the vertically transmitted microbiota consist mainly of plant beneficials and symbionts, and the horizontally acquired microbiota are responsible for the adaptation to the local environment (Bergna et al. 2018). Plant species can differ in the proportions of vertically and horizontally acquired microbes, yet their contribution to the plant microbiome is in a similar range (Abdelfattah et al. 2023), which has been also described for the human microbiome (Hourigan and Dominguez-Bello 2023). However, the composition of the plant microbiota varies during a plant’s life cycle and its assembly is always function-driven (Wicaksono et al. 2021). In addition, the microbiota composition follows specific routes; e.g. flowers and fruits are being mainly colonized from the endosphere (inside plant tissues) but the development of new organs always allows entrance of pathogens as well (Hardoim et al. 2015, Olimi et al. 2022).

What are the functions of the microbiota within the plant holobiont? Plant-associated microorganisms influence the plant already during germination. Certain plant phyla, such as mosses and orchids, are not able to germinate without seed- or soil-derived microbes (Hornschuh et al. 2006). Besides that, microorganisms offer a huge array of beneficial functions for plants. For example, the microbiota are involved in the host plant’s growth, productivity, adaptation, physiology, stress resilience, and immunity. Moreover, the intense interaction with microorganisms has contributed to diversification within the plant kingdom (Van Der Heijden et al. 2008) where more closely related plant species are associated with similar microbial communities; a phenomenon described as phylosymbiosis (Lim and Bordenstein 2020). The symbiotic functional interplay can be explained by plant–microbe coevolution forming diverse holobionts.

Coevolution of holobionts suggests a shared governance between the plant and the microbiome, which is supported by the following observations. First, plant germination and plant growth can depend on hormonal interplay with microorganisms. Plant-associated microorganisms produce a variety of phytohormones (e.g. auxins, cytokinins, and gibberellins), known for their involvement in germination, root growth, and their ability to modify plant root architecture and growth to increase nutrient uptake (Hornschuh et al. 2006, Vacheron et al. 2013). Moreover, they can manipulate stress phytohormone levels, such as plant ethylene levels by 1-aminocyclopropane-1-carboxylate deaminase. The establishment of plant–microbiota relationships requires the exchange of chemical signals and nutrients between the partners. However, the chemical signals involved are largely unknown; plant or microorganism-derived polyamines are one of the important compounds that act as physiological effectors and signal molecules in plant–microbe interactions (Dunn and Becerra-Rivera 2023). Second, plant microbiome assembly and reshuffling is triggered by both, plant and microbes. Recent evidence from genome-wide association studies in plant holobionts revealed host loci with genes involved in plant development, immunity, nutrient uptake, and root exudates that regulate microbiome community structure (Zhang et al. 2023). Third, plant protection towards biotic and abiotic stresses is only achieved by shared governance. Plants have evolved a signaling-mediated stress response to recruit a stress-relieving or protective microbiome, denoted as the ‘cry for help of plant roots’ (Liu et al. 2021, Mendes et al. 2011). Moreover, biocontrol approaches have repeatedly proven that an applied biocontrol agent interacts with both, the host plant (induced immunity) and the microbiome (shifts and depletion of pathogens) (Berg et al. 2021, Pieterse et al. 2014). A fourth example is fruit or flower quality modulation, including flavour, smell, and nutrient contents, which can be a joint venture between plants and their associated microbes that ensures pollination and dispersal. Characteristic flavour compounds of strawberry fruits (Verginer et al. 2010b) and grape berries (Bokulich et al. 2014, Verginer et al. 2010a) were identified to be produced by microbes.

## The plant holobiont in the Anthropocene and implications for one health

Climate change has been identified as a core planetary boundary in the Anthropocene, which has the potential to irreversibly change global systems in the event of insufficient intervention (Lewis and Maslin 2015, Steffen et al. 2015). It is no exaggeration to say that holobionts, plants along with associated microorganisms, largely regulate Earth’s climate and ecosystem response to environmental change. For example, peat mosses of the genus *Sphagnum* store more carbon that any other plant on Earth; the fate of this carbon is uncertain given climate change (van Breemen 1995). Plants are subject of many global change studies, and vegetation changes are obvious all over the world (Humphreys et al. 2019, Yang et al. 2021). In contrast, the impact of anthropogenic factors, the main drivers of current global ecosystem changes, on the microbiome has received less attention. A recent meta-data and literature study suggested a decline in microbial diversity, evenness, and specificity while the whole microbiome shifts into a dysbiotic stage, i.e. characterized by r-strategists and hypermutator prevalence (Berg and Cernava 2022). This corresponds to an increased abundance but reduced diversity of antimicrobial resistance genes, as shown for different built environments with increasing grade of anthropogenic influences (Mahnert et al. 2019). Altogether, the abundance and activity of symbiotic microorganisms appears to be declining, while the number of pathogen outbreaks is increasing. Moreover, Delgado-Baquerizo et al. (2020) provided predictions for the introduction of new pathogens, mainly fungi, into production areas that have remained spared so far. Overall, investigations that incorporate a holistic view of the functional implications of shared governance are missing (Cavicchioli et al. 2019).

While holistic and mechanistic studies on the plant holobiont are rare, initial studies investigating the impact of climate drivers (warming, elevated atmospheric CO_2_) on the plant holobiont show alarming results. The Spruce and Peatland Responses under Changing Environments (SPRUCE; https://mnspruce.ornl.gov/) experiment is a whole-ecosystem warming (+0°C to +9°C) and elevated atmospheric CO_2_ (eCO_2_; +500 ppm) experiment conducted in a regression design to test the impacts of climate drivers on ecosystem response in a *Sphagnum*-dominated ombrotrophic peatland. Results from this experiment indicate that warming causes dysbiosis in the *Sphagnum* phytobiome (i.e. the plant together with its constituent microbiome within the environment), as evidenced by a decline in microbial diversity, a pronounced shift in community composition including diazotrophs, and a decline in nitrogen fixation rates (Carrell et al. 2019). Northern peatlands are often nutrient-poor and thus nitrogen fixation or diazotrophy often comprises a large portion of plant demand for nitrogen in these systems (Salmon et al. 2021, Warren et al. 2017). Diazotrophs of the *Sphagnum* microbiome couple nitrogen fixation to methanotrophy, thereby consuming the potent greenhouse gas methane before it is released to the atmosphere (Kolton et al. 2022, Larmola et al. 2014, Vile et al. 2014). A recent study by Petro et al. (2023) at the SPRUCE site shows that this coupling of the nitrogen and carbon cycles in the *Sphagnum* microbiome is short circuited by climate drivers. Under ambient CO_2_, warming increases plant-available NH_4_-N in surface peat, excess N accumulates in *Sphagnum* tissue, and N_2_ fixation activity decreases. Elevated CO_2_ offsets the effects of warming, which disrupts the accumulation of N in the peat and *Sphagnum* tissues. Methane concentrations in porewater increases with warming, irrespective of CO_2_ treatment. Under +9°C, this results in a 10-fold higher methanotrophic activity in the *Sphagnum* microbiome. Warming’s divergent impacts on diazotrophy and methanotrophy caused these processes to become decoupled at warmer temperatures, as evidenced by declining rates of methane-induced N_2_ fixation and significant losses of keystone microbial taxa. In addition to changes in the *Sphagnum* microbiome, ∼94% mortality of *Sphagnum* was observed between the +0°C and +9°C treatments, possibly due to the collective impacts of warming on N-availability and competition with emerging vascular plant species. Altogether, rising temperatures and atmospheric CO_2_ concentrations are expected to result in a vegetation shift referred to as ‘shrubification’ (Malhotra et al. 2020), whereby shrubs and trees replace *Sphagnum* mosses—the engineers and masters of carbon storage—with significant implications for carbon and nitrogen cycling in boreal peatlands. In parallel, peat decomposition is boosting climate change: as peatland vegetation trends toward increasing vascular plant cover with warming, the massive carbon stores in peatlands are vulnerable to degradation (Hopple et al. 2020) and we can expect a concomitant shift towards increasingly methanogenic conditions and amplified climate–peatland feedbacks (Wilson et al. 2021). In addition, carbon substrate utilization is limited by the availability of terminal electron acceptors and porewater dissolved organic matter, and these controls of microbial peat soil organic matter degradation are dependent on the temperature (Song et al. 2023). Another warming experiment on oak seedlings, carried out under controlled conditions, provided clear evidence that higher temperatures reduce the diversity of oak-inhabiting fungi with influence on the holobiont’s health (Faticov et al. 2021).

Recently, the plant microbiome was included in the one health concept (Flandroy et al. 2018). During the last years, human infections caused by bacterial and fungal pathogens originating from plants has received attention (Berg et al. 2005, Berendonk et al. 2015, Kim et al. 2020). Generally, these microbes can be divided into two groups: human pathogens that cause severe food-borne diseases through association with the plant as an alternative host, and opportunistic pathogens that cause most of the healthcare-associated infections (HCAIs) world-wide (Kim et al. 2020). While the first group is deeply studied and well integrated into food safety concepts, HCAIs of plant or environmental origin are less understood and difficult to avoid (Kim et al. 2020, Yoon and Lee 2018). In general, HCAIs have a severe impact on human health: more than 4 million and 1.7 million patients are affected by HCAIs every year in Europe and in the USA, respectively. Although less so in middle- and low-income countries, in high-income countries, ~30% of ICU patients are affected by at least one episode of HCAI (WHO 2011). HCAIs of plant origin typically occur in immunocompromised, postsurgical, or post-traumatic patients, categorizing them as opportunistic pathogens without specificity (Berg et al. 2005, Kim et al. 2020) but they are often characterized by their high potential for antimicrobial resistance (Berendonk et al. 2015). Antimicrobial resistance is necessary for microbes to colonize plants, especially in the rhizosphere, where antimicrobial compounds are often secreted; under antibiotic pressure in health-care settings, those microbes are selectively enriched (Berg et al. 2005).

The WHO priority list for pathogens with high antimicrobial resistance, which are difficult to control and need urgent attention (WHO 2017), comprise the several bacterial species, which are well-known for their frequent occurrence and positive interaction with plants. In the following list, plant-associated microbes are highlighted in bold: Priority 1: CRITICAL: ***Acinetobacter baumannii, Pseudomonas aeruginosa***, and ***Enterobacteriaceae***, Priority 2: HIGH: ***Enterococcus faecium, Staphylococcus aureus***, *Helicobacter pylori, Campylobacter* spp., *Salmonellae*, and *Neisseria gonorrhoeae*, Priority 3: MEDIUM: *Streptococcus pneumonia, Haemophilus influenza*, and *Shigella* spp. This is confirmed by a systematic analysis for the Global Burden of Disease Study 2019, which identified 33 global bacterial pathogens and five pathogens that were each involved in more than 500 000 deaths in 2019: ***S. aureus, Escherichia coli***, *S. pneumoniae*, ***K. pneumoniae***, and ***P. aeruginosa****. Pseudomonas aeruginosa* is a frequent inhabitant of the rhizosphere, e.g. the rhizosphere of potato, strawberry, oilseed rape, rice, and wheat (Berg et al. 2005), and strains of this species are often selected as excellent biocontrol agents against plant pathogens (Wang et al. 2020, Yasmin et al. 2017). Earlier studies reported potential human pathogens in the rhizosphere of wheat grown on soils with high salt concentration (Egamberdieva et al. 2007). Salt-tolerant *Staphylococcus* species, commonly known as potential human or animal pathogens, have also been observed in the rhizosphere of saltwort (Shurigin et al. 2020). High salt concentrations and soil temperatures in dry areas will foster favourable circumstances for bacteria that have their origins in warm-blooded animals. The family *Enterobacteriaceae* is a specific group which colonize above-ground plant parts and inner compartments of (healthy) seeds (Lindow and Brandl 2003, Rossmann et al. 2012, Simonin et al. 2022). It was observed that the intraspecific diversity of *Enterobacteriaceae* in pumpkin seeds is a health indicator for seed and plants in the field (Adam et al. 2018). These examples show that emerging human pathogens with antimicrobial resistance profiles can be associated to plants. Several of them are known for their plant-beneficial interaction, and are thus of interest for biotechnological applications. The functions and ambivalent interactions with different hosts must be studied intensively and application or commercialization of potential (opportunistic) human pathogens should be carefully assessed or restricted.

It is imperative to thoroughly analyze another significant aspect, namely the overlap of bacterial taxa found in plants that possess the potential to cause infections in humans. This examination will determine if these taxa possess identical characteristics across both domains. The plant microbiome can act as reservoir for opportunistic, often multiresistant pathogens. This can be explained by the ecology of the plant holobiont and its sessile lifestyle producing secondary metabolites with antimicrobial activity. For successful plant colonization of an endophyte, all steps—recognition, adherence, invasion, and establishment—are required; the same are required by pathogens (Berg et al. 2005). Several bacterial genera, including *Burkholderia, Enterobacter, Pseudomonas, Ralstonia, Staphylococcus*, and *Stenotrophomonas*, contain plant-associated strains that can have different effects on both plants and humans. (Berg et al. 2005). While opportunistic bacteria from plants do have some properties in common, each of these opportunistic pathogens has its own features, summarized, e.g. for *P. aeruginosa* (Laborda et al. 2022) and *Stenotrophomonas maltophilia* (Lira et al. 2017). Another example is *Shigella;* recently, human-pathogenic bacteria *Shigella boydii* and *S. flexneri* were found to colonize leaves and roots of *Arabidopsis*. Further studies revealed that the *Shigella* type III secretion system not only regulates the pathogenesis of shigellosis in humans but also plays a central role in bacterial proliferation in the plant, for which the immunosuppressive activity of two type III effectors, OspF and OspG, was required (Jo et al. 2019). Although bacteria are considered as major burden, there are also examples for fungal pathogens. The fungus *Chondrostereum purpureum*, known to cause Silver leaf disease of plants, particularly of the rose family, was previously not considered a threat to humans. However, a recent report has documented the first case of a paratracheal abscess in humans caused by *C. purpureum* (Dutta and Ray 2023). Altogether, viral, bacterial, and fungal pathogens of plants and animals can adapt to both abiotic and biotic factors of the Anthropocene, including climate change (Cavicchioli et al. 2019, Giraud et al. 2017). Moreover, agricultural ecosystems share global features, and the dispersal of pathogens through human travel and plant material transport enhances the potential of pathogens to broaden both, the environmental and the host range, even cross-kingdom, and increase their chances to enter hospital environments. The molecular mechanisms behind cross-kingdom infections remain unclear, but should be studied in detail to determine mechanisms for disease prevention (Kim et al. 2020).

While the biodiversity hypothesis/missing microbe theory is well-established in the context of human health (Blaser 2017, Finlay et al. 2021, Hanski et al. 2012), plant health has received less attention in this regard. However, the transformation of the plant microbiome in the Anthropocene, i.e. declined microbial diversity, evenness, and specificity, and increased abundance of r-strategists, hypermutators, and antimicrobial resistance, indicates that plant health is already being remarkably affected. Microbiome-based control concepts are an interesting alternative to current pesticides, fertilizers and disinfectants (Vandini et al. 2014). Plant and environmental health are directly linked to human health which becomes clear from the unintended consequences of intense agriculture in the former Aral Sea region (Wicaksono et al. 2023b) and destructive mining practices in the Salares of the hyperarid Atacama Desert in Northern Chile (Bonelli and Dorador 2021). Another link between human health and the depleted plant microbiome, especially the “edible microbiome”, was already suggested in 2015, which needs further evaluation (Berg et al. 2015, Wicaksono et al. 2023a).

## Conclusions

In the last two decades of microbiome research, the holobiont theory and the hologenome theory of evolution were confirmed, thereby revealing new insights into coevolution and shared governance of eucaryotic hosts and their associated microbiomes. Here, we describe the implications for plant holobionts and their response to ongoing global environmental changes. Importantly, they are integrated into ecosystems and interact with other holobionts, designated as holobiomes or meta-communities (Bragina et al. 2015). Holobiome is a term used for different purposes but, according to the origin of the word it combines micro- and macrobiomes into the holobiome [Holobiome. The words “holo” and “biome” are of Ancient Greek origin. “Holo” Hólos <όλος> means whole, while the term “biome” is composed of the Greek word bíos (βιος, life) and modified by the ending “ome” (Anglicization of Greek)]. However, anthropogenic activities have changed the signature of the environmental and host-associated microbiomes, resulting in severe health problems in the holobiome. Action is needed to protect plant holobionts as the centerpieces of the holobiome, with major implications for human and environmental health on a rapidly changing Earth.

Holobiont research catalyzes new approaches and a rethinking of current practices. Here, we provide the following examples:

- The role of mycorrhizal fungi symbionts as evolutionary drivers of plants’ colonization of land ecosystems and diversification is well-established but less is known about the other members of the plant microbiome during evolution—understanding can boost microbiome-supported agriculture.
- Breeding shifted the plant microbiome to a similar extent than the crop phenotype. Microbiome-based breeding can maintain the symbiotic and beneficial part of the plant microbiome. Here, understanding the physiology and harnessing the functional potential of the seed microbiome will help to develop sustainable approaches and provide crucial insights into plant microbiome coevolution.
- Vertical transmission and horizontal acquisition of the plant microbiome are crucial for holobiont’s resilience. Microbiome-based plant protection strategies can be developed to avoid outbreaks of pathogens and pests.
- A healthy plant is characterized by a microbiome, i.e. diverse in structure and function; functional diversity matters. Instead of reducing microbial diversity, e.g. by seed treatments and in agriculture in general, new approaches that enrich microbial diversity should be implemented. Microbiome management by abiotic (organic materials and humic acids) and biotic (microbial seed coatings, transplants, and biocontrol) treatments can help to develop resilient plants.
- The plant microbiome plays a crucial role in the one health concept, as reservoir for food pathogens as well as opportunistic, often multiresistant pathogens that cause HCAIs. Especially for the latter, new microbiome-based control concepts are essential. Certainly, new antibiotics and disinfectants are very important in hospital environments and disease treatments; in the long-term and on environmental scale, we must rethink current practices.
- Plants with its diverse coevolved plant microbiome is crucial for planetary health. Plant holobionts drive global biogeochemical cycles and mediate planetary resilience in the face of massive and ongoing environmental changes. The adaptive management and restoration of ecosystems in response to global change should include holobiont-based strategies.
- Interdisciplinary and holistic approaches that consider shared governance as a principle for all holobionts are inevitable to save the planet.

Our joint discussion identified the following knowledge gaps:

- The structure and function of the holobiont is well-studied but communication and interaction between the host and the microorganisms as well as the entire microbiome is less understood. Inter- and intraspecific communications by volatile organic compounds, small molecules, membrane vesicles (exosome), or sound vibration represent particularly intriguing areas that warrant further research.
- Viruses are drivers of the microbiome evolution. At the same time, they are an often neglected component, which needs more attention.
- Holistic approaches should also include information from southern hemisphere ecosystems, which are less represented in the literature.
- The one health concept is based on interconnected holobionts. While transmission of pathogens between different hosts and environments is well-investigated, less is known for beneficial microorganisms.
- The exposome can be defined as the measure of all (physical, biological, and chemical) exposures of an individual in a lifetime and how those exposures relate to health. The plant and environmental microbiome is not yet considered in the concept but must be implemented as well. Especially food as microbial transmission route to the human gut (“the edible microbiome”) needs further studies.
- Nonfood plant holobionts that drive the global carbon cycle and provide critical ecosystem services, are severely understudied.
- Genotype–phenotype relationships are not understood at the holobiome scale.
- We need to advance understanding of how microbiomes control the physiological ecology of plant holobionts at the ecosystem scale (cotranscriptomics).
